# Supplementary material for: Insights and inspirations: A qualitative exploration of community health workers’ motivations in Myanmar and Bangladesh
Source: PLOS Glob Public Health. 2024 Oct 10;4(10):e0003773. doi: 10.1371/journal.pgph.0003773 (PMC11466398; doi:10.1371/journal.pgph.0003773)
Supplement: S2 File — (PDF) [file pgph.0003773.s002.pdf]

Key Informant Interview Guide

1. Please share your position, your roles in the organization, your years of experience working with the community health workers and your years of experience working in the non-profit field.
   Probes:
   a. What were/are organizations you have worked for?
   (for each one if there are more than one organization)
   b. What is your position there?
   c. What kind of responsibilities do you have?
   d. How many years have you worked there?

2. How does your organization/organizations recruit new community health workers?
   Probes:
   a. Please explain the process.
   b. What are the challenges you have faced to recruit new CHW?
   c. What are the things that work well to recruit new CHW?

3. Please tell me about the issues your organization has with retention of CHWs.
   Probe:
   (for each issue)
   a. In your opinion, which factors are influencing it?

4. What are the challenges you have faced in supporting and/or training community health workers?
   Probes:
   (for each challenge)
   a. How did you overcome this challenge?
   b. What are the things that work/didn't work to overcome this challenge?

5. If CHWs have questions about their work or challenges, who do they go to for help?
   Probes:
   a. Where or from whom can they get support?
   b. In what way? How did they usually get help?
   c. If they cannot reach them or the issues are not resolved, what can they do as a next step?

6. What are additional challenges for your organization in supporting CHWs after the COVID-19 outbreak? Please share details of your experience.
   Probes:
   (for each challenge)
   a. How did you overcome this challenge?
   b. What are the things that work/didn't work?

7. Does the Feb 2021 coup affect your organization in supporting CHWs in any ways? If so, please explain how? How did you handle them?

Probes:
(for each challenge)
  a. How did you overcome this challenge?
  b. What are the things that work/didn't work?

8. What kind of additional support do you need to help your community health workers better?
   Probes:
   (for each one)
   a. Where or who can support this one?
   b. How do you think you can get it? Can you think of any possible way?

9. What will you recommend donor/international support do/change to better support CHWs in your organization? Please elaborate your thoughts.
   Probe:
   (for each one)
   a. Why?
   b. How can they do that?

10. Can you please recommend community health workers whom I should interview for their life stories?
    Probe:
    (for each one)
    a. Please share name, position, and organization of the CHW
    b. Why do you recommend this person?
    c. What are the things I need to be sensitive and careful during the interview process?
    d. Can they speak Myanmar Language? Will they need an interpreter? If so, which language do they speak?
    e. How can I contact him/her? (Phone number, email, etc.,)

11. Are there any other things you would like to add or say? Are there any things you would like to ask me?
