# Supplementary material for: Insights and inspirations: A qualitative exploration of community health workers’ motivations in Myanmar and Bangladesh
Source: PLOS Glob Public Health. 2024 Oct 10;4(10):e0003773. doi: 10.1371/journal.pgph.0003773 (PMC11466398; doi:10.1371/journal.pgph.0003773)
Supplement: S3 File — (PDF) [file pgph.0003773.s003.pdf]

Life Story Interview Guide

1. Please tell me about your age, marital status, ethnicity, educational background and family background?
   Probes:
   a. Have you worked in other organizations before? If so, please share your title, responsibilities and how many years you have worked there.
   b. Why did you leave your former organization?

2. Could you please draw a lifeline like this to reflect your life from the beginning to the current situation? Please add good times and good situations above the horizontal line and bad times and bad situations below the horizontal line. Please also think and add your journey as a community health worker and include memorable good times and bad times and significant changes there. Please take your time in drawing. (about 15-30 minutes)

An example of a lifeline drawing

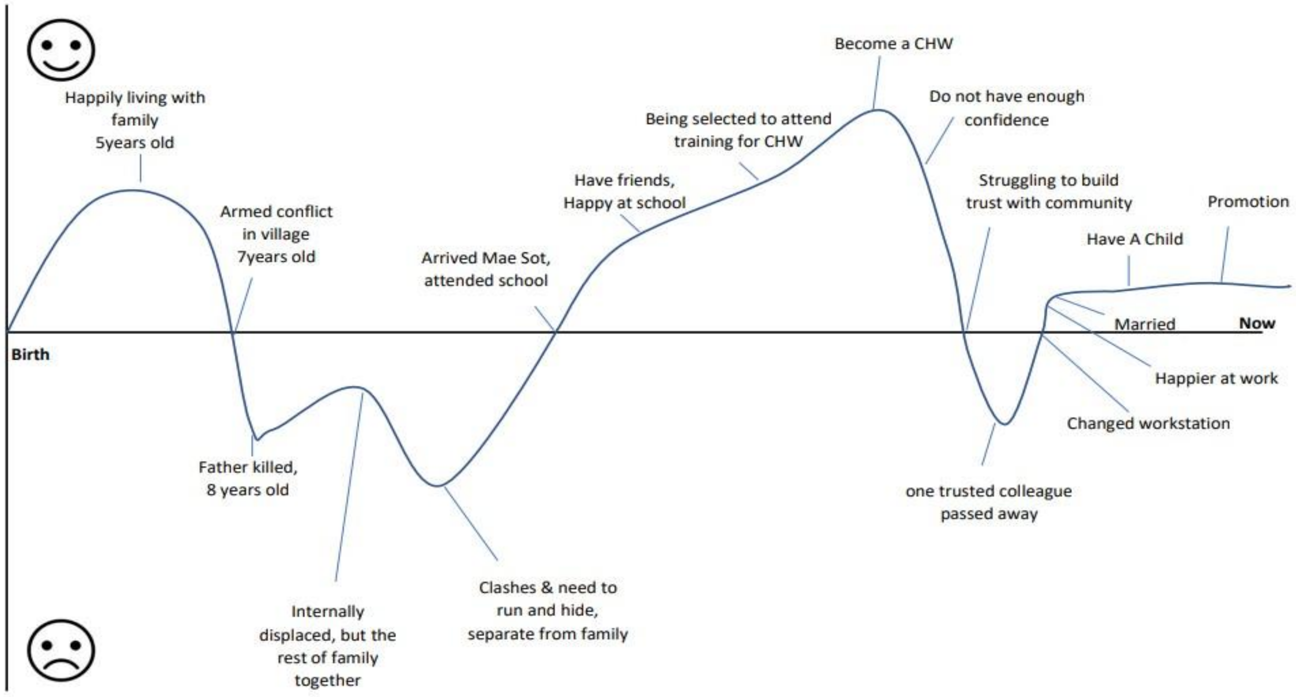

(Continue the interview only after the drawing is finished completely.)
Please explain your lifeline drawing to me in detail and walk me through the events there?
Probes:

a. What happened there?
b. How do you feel about that?
c. What happened after that?
d. How did you overcome that?
e. Can you please tell me a bit more about that?

3. Please share how you came to be a health worker?
   Probes:
   a. What were the things that motivated you to start?
   b. What were/are the things that motivate you to keep on going?

4. How was your experience as a health worker similar or different from your expectations of what the work would be like?

5. How do you think your community views your role?
   Probes:
   a. Is it because of your skills or links to EAO or EHO?
   b. How does this make you feel?

6. To what extent do you feel your role in the community is respected? Why?

7. What are the most inspiring moments in your work?
   Probe:
   (until they do not list any more)
   a. Are there any other moments?

8. What are the most disappointing moments in your work?
   Probe:
   (until they do not list any more)
   a. Are there any other moments?

9. What made you stay in this job?
   Probe:
   (until they do not list any more)
   a. Are there any others?

10. What are your ambitions in your life?

11. What are your goals in this work?
    Probe:
    a. How are they different from or relevant to your ambitions in life?

12. Please tell me about some questions you've had about your work or challenges you've faced. When you have questions about your work or challenges, who do you go to for help?
    Probe for each person:

   a. How helpful are they?

Probe for each question/ challenge:

   a. How did you overcome it?

13. What are the changes you would like to make with your job/ responsibilities if you have the power to do so? Why?
14. What are the changes you would like to make with your organization's support if you have the power to do so? Why?
15. What work-related challenges have you faced that are specifically because of COVID-19? Please elaborate your experience.
    Probes:
    (for each challenge)
    a. How did you overcome this challenge?
    b. What are the things that work/didn't work?
16. How has the Feb 2021 coup affected your work? Please elaborate your experience.
    Probes:
    (for each challenge)
    a. How did you overcome this challenge?
    b. What are the things that work/didn't work?
17. What kind of additional support/training do you want to have as a community health worker? Why?
18. What will you recommend donor/international support do/change to better support CHWs? Why? Please express your thoughts in detail.
19. Are there any other things you would like to add or say? Are there any things you would like to ask me?
