# Supplementary material for: Insights and inspirations: A qualitative exploration of community health workers’ motivations in Myanmar and Bangladesh
Source: PLOS Glob Public Health. 2024 Oct 10;4(10):e0003773. doi: 10.1371/journal.pgph.0003773 (PMC11466398; doi:10.1371/journal.pgph.0003773)
Supplement: S4 File — (PDF) [file pgph.0003773.s004.pdf]

A Qualitative Insight into the Motivations of Community Health Workers in Myanmar's Conflict-Affected Areas and a Marginalized Community in Bangladesh

Authors: Nyo Yamonn, Catherine Lee, Tom Traill

Background:

Despite the recognized roles and potential of Community Health Workers (CHWs) in various settings, there is limited literature on CHWs from Myanmar who serve conflict-affected and marginalized populations. These CHWs are integral to the health system networks of Ethnic Health Organizations in conflict-ridden regions of Myanmar. Similarly, in Cox's Bazar, Bangladesh, where nearly a million Rohingya refugees from Myanmar reside, CHWs play a crucial role in overcoming language, cultural, and belief barriers to healthcare for the Rohingya. This study aims to enhance our understanding of the unique challenges these CHWs face through their firsthand experiences, thereby contributing to the improvement of healthcare delivery in these regions.

Methods:

Thirty-four life story interviews with lifeline tool as an added element were conducted with CHWs from conflict-affected regions in southern Shan State and Kayin State of Myanmar, as well as those in Cox's Bazar camps in Bangladesh. Additionally, eight key informant interviews were conducted with individuals in leadership positions from organizations that work with or provide training to CHWs. Thematic analysis, facilitated by NVivo 14 software, and a condensed Socio-Ecological Model were employed for comprehensive data analysis.

Results:

Our findings underscore that CHWs are primarily motivated to join organizations by the prospect of acquiring skills and knowledge. In the Bangladesh study area, the focus was on job-related skills and health knowledge, while in Kayin and southern Shan study areas, healthcare skills and knowledge took precedence. Remuneration, though inadequate to meet basic needs except in the Bangladesh study area, was identified as crucial for retention, aligning with existing literature. Feeling valued by the community emerged as a significant retention factor, linked to relationships with community members and leaders. Mental health support emerged as a potential need for CHWs. Funding deficits and fragmented support contributed to organizational challenges, affecting various aspects of CHW programs and retention.

Conclusions:

Ensuring the effectiveness and sustainability of CHW programs in conflict-affected and marginalized regions necessitates a fundamental shift towards supporting the overall needs of organizations and health systems with a long-term commitment. This includes a focus on CHWs' mental health and the engagement of stakeholders, including CHWs themselves. A reliance on short-term and fragmented solutions may lead to a reversion to pre-existing situations once these solutions are removed. Sustainability planning is paramount to breaking the cycle of CHW turnover and maximizing previous investments in these challenging contexts.
