# Supplementary material for: Insights and inspirations: A qualitative exploration of community health workers’ motivations in Myanmar and Bangladesh
Source: PLOS Glob Public Health. 2024 Oct 10;4(10):e0003773. doi: 10.1371/journal.pgph.0003773 (PMC11466398; doi:10.1371/journal.pgph.0003773)
Supplement: Attachment — Submitted filename: Response to Reviewers.docx [file pgph.0003773.s005.docx]

**Response to Reviewers**

Journal Requirements:

1. Please include a complete copy of PLOS’ questionnaire on inclusivity in global research in your revised manuscript. Our policy for research in this area aims to improve transparency in the reporting of research performed outside of researchers’ own country or community. The policy applies to researchers who have travelled to a different country to conduct research, research with Indigenous populations or their lands, and research on cultural artefacts. The questionnaire can also be requested at the journal’s discretion for any other submissions, even if these conditions are not met. Please find more information on the policy and a link to download a blank copy of the questionnaire here: https://journals.plos.org/globalpublichealth/s/best-practices-in-research-reporting. Please upload a completed version of your questionnaire as Supporting Information when you resubmit your manuscript.

**The questionnaire is completed and will be uploaded with the resubmitted manuscript.**

2. Your current Financial Disclosure states, “The study is funded by Community Partners International's Core Funds.” However you do not provide a Funding information on the online submission form. Please indicate by return email the full and correct funding information for your study and confirm the order in which funding contributions should appear. Please be sure to indicate whether the funders played any role in the study design, data collection and analysis, decision to publish, or preparation of the manuscript.

**The correct funding information should appear as: 'The study is funded by Community Partners International's Core Funds.' The funders were not involved in any part of the study or the preparation of the manuscript.**

3. Please provide separate figure files in .tif or .eps format.

For more information about figure files please see our guidelines:

https://journals.plos.org/globalpublichealth/s/figures

https://journals.plos.org/globalpublichealth/s/figures#loc-file-requirements

**Yes, the figures will be resubmitted in the correct format according to the guidelines.**

4. In the online submission form, you indicated that "The datasets used and analyzed during the current study are available from the corresponding author on reasonable request. The data cannot be shared publicly due to data sensitivity.".

All PLOS journals now require all data underlying the findings described in their manuscript to be freely available to other researchers, either

a. In a public repository,

b. Within the manuscript itself, or

c. Uploaded as supplementary information.

This policy applies to all data except where public deposition would breach compliance with the protocol approved by your research ethics board. If your data cannot be made publicly available for ethical or legal reasons (e.g., public availability would compromise patient privacy), please explain your reasons by return email and your exemption request will be escalated to the editor for approval. Your exemption request will be handled independently and will not hold up the peer review process but will need to be resolved should your manuscript be accepted for publication. One of the Editorial team will then be in touch if there are any issues.

**The data was collected from vulnerable and sensitive populations across three study areas, including participants from the Rohingya population in Bangladesh and the Kayin and Shan ethnic groups from areas controlled by armed groups in Myanmar. In our protocol, approved by our research ethics board, we assured participants of the confidentiality of their data. The approval letter, which includes the address of the research ethics board, along with our informed consent forms, is attached to this submission.**

**Additionally, the raw data cannot be made publicly available due to security concerns related to our organization's work in the study countries. If required, we can privately submit the de-identified raw data to the editors, with a request that it not be made publicly accessible. The manuscript already includes detailed thematic summaries, providing a comprehensive overview of our findings. Requests for raw data can be directed to the Chief Executive Director of our organization, Dr. Si Thura, at** [**sithura@cpintl.org**](mailto:sithura@cpintl.org)**.**

5. Image.1.pdf: Please confirm (a) that you are the photographer; or (b) provide written permission from the photographer to publish the photo(s) under our CC-BY 4.0 license.

**Yes, I received approval from the photographer, and her written approval email will be uploaded along with the resubmitted manuscript.**

Additional Editor Comments (if provided):

The authors report a qualitative study that explores motivations and retention of CHWs in conflict prone areas in Myanmar and the Rohingya camps in Bangladesh. This is a crucial area of inquiry because health in conflict prone areas, politically unstable areas and refugee camps can be precarious and health workers play a major role in ensuring basic health in these areas. Their wellbeing, support systems and coping strategies are not studied adequately. Findings of such a study can help understand the situation of CHWs in conflict zones and in refugee camps.

**Due to manuscript length constraints, some areas were omitted. We are currently conducting a follow-up study focusing on the mental health support needs of CHWs, delving deeper into their wellbeing, support systems, and coping strategies.**

The authors have provided very good description of the study settings in Myanmar and the Cox Bazar Rohingya refugee camp of Bangladesh. The exact details of who did the lifeline interviews, who did the lifeline visualizations, how and where they were done must be provided in detail. Reflexivity of the researchers and interviewers must also be provided to understand the process of the qualitative inquiry in detail.

**We have provided the requested details in the revised manuscript page 10. The lifeline visualizations were created by participants, while interviews were conducted by trained local data collectors in private, familiar settings. These data collectors, fluent in the local languages, received guidance on maintaining self-awareness and minimizing potential biases during the research process. Additionally, the team held regular debriefing sessions where data collectors could discuss their experiences, challenges, and any emotional responses, which contributed to refining the interview process.**

Some more details of how the study participants were selected must be provided. How were these participant identified? Who identified them?

**We have provided the requested details in the revised manuscript page 8. In the early stage of the study, focal points at each organization were provided with specific criteria on the characteristics of CHWs we aimed to interview, including type and level of work, years of experience, gender, and assigned area. These focal points then facilitated the process by making initial contact with potential participants. As the study progressed, data collectors obtained authorization from most organizations to communicate directly with potential participants. Consequently, the data collectors approached individuals from these organizations directly to request their participation in interviews. KII participants were identified by the study team and contacted directly by the principal investigator.**

There is a good description about the study team in terms of the training provided to them. It would be useful to describe characteristics of the study team, their expertise, their level of experience and personal traits that might have an influence on the data collection, analysis and interpretation.

**We have provided the requested details in the revised manuscript page 8. The study team included the principal investigator, a native of Myanmar with strong local knowledge and community health expertise, along with some qualitative research experience. The data collectors, proficient in local languages and experienced in qualitative data collection, were familiar with their respective study areas in Myanmar and Bangladesh. Additionally, experienced researchers provided valuable input on research design, methodology, and data analysis. The team’s combined expertise in the local context and qualitative data collection ensured effective and culturally sensitive data gathering.**

Was an interview checklist used? What probes were used to explore the lifelines? What materials were provided to sketch the lifeline? Did all participants draw their lifelines? Did any of them request support for drawing?

**We have provided the requested details in the revised manuscript page 9 and 10. Yes, the interview checklist and probes used for exploring the lifelines will be submitted along with the revised manuscript. Participants were provided with A4 papers and pens or pencils for drawing their lifelines, and sample drawings were also provided. Although adherence to instructions and levels of participation varied, most participants completed their drawings independently without requesting additional support.**

One of the researchers has coded and re-coded the data totally 3 times. The authors should provide details on which of them did the analysis? What are their characteristics? If they found a need to modify their code during subsequent coding sessions, how did they reconcile the difference?

**We have provided the requested details in the revised manuscript page 11. The principal investigator, selected for her availability, bilingual abilities, and understanding of the research contexts, conducted the coding and re-coding process. Differences identified during the iterative coding sessions were reconciled by revisiting the data and refining the coding scheme to better capture the nuances of the data. These steps have been detailed to clarify the process and enhance the transparency of our analysis.**

Are names of the CHWs original or have they been changed to protect confidentiality?

**The names provided are pseudonyms, not their real names, to protect their confidentiality.**

Reviewers' comments:

5. Review Comments to the Author

Reviewer #1: You have presented a well-structured protocol. I have a few suggestions:

1. Include more specific examples or case studies where similar methodologies have been successfully applied to enhance the justification of the approach. It will strengthen validation.

**Address in page 28**

2. Address any potential limitations regarding the data collection process in conflict-affected regions, such as logistical challenges or data quality issues. Many can learn from this.

**Address in page 33**
